# DANCE AS A CREATIVE ARTS PROGRAM FOR SEINOR ADULTS WITH PARKINSON’S DISEASE AND THEIR CAREGIVERS

**DOI:** 10.1093/geroni/igae098.2238

**Published:** 2024-12-31

**Authors:** LaVona Traywick

**Affiliations:** Arkansas Colleges of Health Education, Fort Smith, Arkansas, United States

## Abstract

The Dance with PD (Parkinson’s Disease) program is offered as a free community creative arts outreach program by Arkansas Colleges of Health Education (ACHE), Research Institute Health & Wellness Center for individuals with PD and their care partners. Dance with PD is a therapeutic approach to programming that involves dance and movements as a form of therapy for individuals living with Parkinson’s Disease. The program aims to improve mobility, balance, coordination, and overall quality of life through structured dance exercises and routines. The program is offered by a trained instructor and the sessions are tailored to the specific needs and abilities of the participants. Volunteer assistants are students from ACHE’s medical college. The class is offered once a week for one hour to mimic a traditional dance class. Due to the rolling admission, participants have attended for different lengths of time when they complete their assessments. The Dynamic Gait Index (DGI), Falls Efficacy Scale, and Geriatric Depression Scale—short form are administered each semester and compared to attendance records.

